# Searching for Cellular Partners of Hantaviral Nonstructural Protein NSs: Y2H Screening of Mouse cDNA Library and Analysis of Cellular Interactome

**DOI:** 10.1371/journal.pone.0034307

**Published:** 2012-04-10

**Authors:** Tuomas Rönnberg, Kirsi Jääskeläinen, Guillaume Blot, Ville Parviainen, Antti Vaheri, Risto Renkonen, Michele Bouloy, Alexander Plyusnin

**Affiliations:** 1 Department of Virology, Haartman Institute, University of Helsinki, Helsinki, Finland; 2 Transplantation Laboratory, Haartman Institute, University of Helsinki, Helsinki, Finland; 3 Research Programs Unit, Infection Biology Research Program, Haartman Institute, University of Helsinki, Finland; 4 Bunyaviridae Unit, Pasteur Institute, Paris, France; The Scripps Research Institute, United States of America

## Abstract

Hantaviruses (*Bunyaviridae*) are negative-strand RNA viruses with a tripartite genome. The small (S) segment encodes the nucleocapsid protein and, in some hantaviruses, also the nonstructural protein (NSs). The aim of this study was to find potential cellular partners for the hantaviral NSs protein. Toward this aim, yeast two-hybrid (Y2H) screening of mouse cDNA library was performed followed by a search for potential NSs protein counterparts via analyzing a cellular interactome. The resulting interaction network was shown to form logical, clustered structures. Furthermore, several potential binding partners for the NSs protein, for instance ACBD3, were identified and, to prove the principle, interaction between NSs and ACBD3 proteins was demonstrated biochemically.

## Introduction

Hantaviruses constitute the *Hantavirus* genus in the family *Bunyaviridae* [1]. Natural hosts for hantaviruses are mostly Muroidea rodents of *Muridae* and *Cricetidae* families (for the current rodent taxonomy, see [2]) and insectivores of family *Soricidae*. Very little is known about insectivore-borne hantaviruses, and the focus of this study is exclusively on rodent-borne hantaviruses. When transmitted to humans, hantaviruses cause hemorrhagic fever with renal syndrome (HFRS) or hantavirus (cardio) pulmonary syndrome (HPS) [3], [4].

Hantaviruses are negative-strand RNA viruses (NSRV) with a tripartite genome consisting of Large (L), Medium (M) and Small (S) segments [1], [4], [5]. The L segment encodes the viral RNA-dependent RNA polymerase (L protein). The M segment encodes two surface glycoproteins Gn and Gc. The S segment encodes the nucleocapsid (N) protein. In some hantaviruses the S segment also has an open reading frame (ORF) for a nonstructural protein (NSs) [5]. Using Tula virus (TULV) and Puumala virus (PUUV) as models it was shown that the NSs ORF is functional and the product interferes with interferon (IFN) production by inhibiting the activity of IFN-β promoter and also NF-kB- and IRF-3-responsive promoters [6]. Importantly, the expression of full-length NSs protein increases the survival time of TULV in interferon-competent cells [7]. Recently it was shown that the NSs protein of TULV accumulates in the perinuclear area in infected and transfected cells [8]. Functions of the hantaviral NSs protein other than IFN inhibition (e.g., regulation of viral transcription/replication or RNA-binding) remain unknown.

Several members of the family *Bunyaviridae* encode NSs proteins. Similar to hantaviruses, orthobunyaviruses express their NSs proteins from an ORF overlapping the N-coding sequence in the S-segment. The NSs protein of phleboviruses and tospoviruses are encoded using the ambisense strategy. Nairoviruses do not encode an NSs protein [1]. In the genus *Orthobunyavirus*, known human pathogens such as Bunyamwera virus (BUNV) and La Crosse virus (LACV) both express an NSs protein which helps to evade host immune response(s) by interfering with interferon production [9], [10]. In BUNV the NSs protein inhibits directly mRNA production by binding to the transcription complex. Interestingly, in LACV the interferon-suppressive effect is seen only in mammalian cells where it suppresses the RNA interference (RNAi) mechanism [11]. In insect cells these effects are not observed [10]. The Rift Valley fever virus (RVFV) of the genus *Phlebovirus* employs a similar strategy to silence host interferon response [12]. In addition the NSs protein of RVFV specifically downregulates expression of double-stranded RNA-dependent protein kinase (PKR), an essential component of the innate immunity [13]. It has been also reported that the NSs protein of RVFV is associated with gamma satellite pericentromeric DNA and induces chromosomal segregation defects [14]. It is thought that these effects contribute to the pathogenicity of RVFV. In the case of the Uukuniemi virus (UUKV), which is considered non-pathogenic [15], the function(s) of its NSs remain unknown. However, this suggests that the NSs protein is not the sole factor that influences the pathogenicity of a virus. UUKV NSs seems to associate with the 40 S ribosomal subunit of host [16], but is not required for viral replication [17]. The NSs protein of tospoviruses is involved in evading of siRNA-mediated antiviral gene silencing [18].

Other NSRVs also encode nonstructural proteins. Probably the best known is the NS1 protein of influenza viruses (family *Orthomyxoviridae*), which is a multifunctional protein involved in host immune response suppression and viral replication and pathogenesis [19]. Although the exact mechanisms of NS1 vary between virus strains [19], it commonly interacts with host innate immunity and prevents IFN-β production. More specifically, NS1 is known to bind to both viral RNA, to mask it from recognition by pattern recognition receptors (PRRs), and directly to PRRs (such as RIG-I) hindering their function [20]. It also binds to the PKR and other cellular antiviral proteins [19]. In addition, NS1 can inhibit transcription on general level by preventing polyadenylation of host mRNA [20] or post-translational processing of RNA polymerase II (RNAPII) [19]. Recently it has been shown that NS1 interferes with the host RNAi machinery [21], but its significance for virus pathogenicity is still unclear.

The host cell partners of the BUNV and RVFV NSs proteins have been best characterized. BUNV NSs protein associates directly with MED8, a component of the Mediator coactivation complex which regulates activity of RNAPII [9]. As a result RNAPII is degraded resulting in a general disruption of host cell transcription. It has also been reported that the interaction with MED8 alone is not enough to cause the RNAPII degradation, and that the BUNV NSs protein has additional, as yet unidentified function(s) [22]. The RVFV NSs protein affects the RNAPII activity as well, but employs a different mechanism. It binds to the p44 subunit of the general transcription factor TFIIH [23]. The p44 subunit is sequestered to the nuclear NSs-containing filamentous structures thus decreasing the concentration of a functional TFIIH complex and reducing the overall host transcription [23]. Additionally, the NSs protein recruits to these nuclear filaments the SAP30 protein, which associates with YY1 and other corepressor/coactivator factors [24]. YY1 is an important factor for regulating virus-induced IFN-β transcription [25], and its recruitment to nuclear filaments through SAP30 specifically inhibits IFN-β expression. The RVFV NSs protein also downregulates expression of the PKR post-transcriptionally [12], but the mechanism is not known yet. It has been suggested that the NSs protein interacts with E3 ubiquitin ligase and targets the PKR for proteasomal degradation [13].

Bunyaviral NSs proteins are involved in other virus-host interactions as well. As mentioned above, LACV NSs counteracts the effects of short interfering RNA (siRNA)-mediated gene silencing [11], but the exact mechanism remains known. In a similar fashion, the NSs protein of tomato spotted wilt virus (TSWV) of the genus *Tospovirus* binds to both siRNA and long double-stranded RNA (dsRNA), inhibiting the Dicer-complex- mediated dsRNA cleavage and thus evading antiviral gene silencing [18], [26].

The aim of this study was to find potential cellular partners for the hantaviral NSs protein. Toward this aim, yeast two-hybrid (Y2H) screening of mouse cDNA library was first performed followed by a search for potential NSs protein counterparts via analyzing a cellular interactome.

## Materials and Methods

### Plasmids

NSs ORFs of PUUV, strain Sotkamo [27] and TULV, strain Moravia 5302 Ma/94 [28] were cloned into the “bait”-plasmid pGBKT7 (Clontech, Mountain View, CA).

### The Y2H assay

To test that the NSs baits do not autonomously activate the reporter genes in the yeast strain in the absence of a prey protein and do not induce toxicity, the yeast strain AH109 (Clontech, California, USA) was co-transformed with empty pACT2 and either the pGBKT7-PUUV NSs construct or pGBKT7-TULV NSs plasmid, then plated on SD/-Leu/-Trp plates and incubated at +30°C for 3 days. From these yeast cultures, the colonies were inoculated into 3 ml of SD/-Leu/-Trp liquid medium and incubated at +30°C overnight under agitation (220 rpm), then the samples were cultured on SD/-Leu/-Trp and SD/-Leu/-Trp/-His/-Ade plates. The plates were incubated at +30°C for 3–5 days.

To verify that NSs proteins are expressed from yeasts transformed with their respectives plasmids, the colonies from SD/-Leu/-Trp plates containing the following samples: (1) empty pGBKT7 - empty pACT2, (2) pGBKT7-PUUV NSs - empty pACT2, and (3) pGBKT7-TULV NSs - empty pACT2 were transferred to SD/-Leu/-Trp liquid medium and incubated at +30°C on a shaker overnight. From these, the protein extracts were prepared using urea/SDS (Clontech's Yeast Protocols Handbook 14.3.2001). Proteins were detected by immunoblotting using anti-c-myc mouse monoclonal antibody (Roche, Basel, Switzerland).

To estimate the cDNA library titer, mouse 17-day Embryo Matchmaker cDNA Library in pACT2 pre-transformed in Y187 yeast strain (Clontech, Mountain View, CA) was diluted to 1∶100, 1∶1000, 1∶10 000 and 1∶100 000 with YPD medium. 100 µl of mixture was cultured on SD/-Leu plates, and the plates were incubated at +30°C for 3–5 days.

To screen the library, 2 µl of either PUUV NSs-pGBKT7 or TULV NSs-pGBKT7 plasmid was added to 4 µl of salmon sperm DNA (10 mg/ml, heated at 95°C for 10 min), 150 µl of the yeast strain AH109 cells and 400 µl of 50% PEG_3350_ in 0.1 M LiAc in TE. The mixture was incubated at +30°C for 30 min and +42°C for 25 min. Yeasts were washed twice with 0.5 ml YPD, and resuspended in 200 µl YPD. The mixture was spreaded on SD/-Trp plates, and incubated at +30°C for 3–5 days.

PUUV NSs-pGBKT7- or TULV NSs-pGBKT7- transformed AH109 yeast colonies from SD/-Trp plates were cultivated into 50 ml of SD/-Trp medium, and incubated at +30°C on a shaker for 18 h, and 21.5 h, respectively. Cells were collected by centrifugation at 1000×g during 5 min and resuspended into 5 ml SD/-Trp medium. To the mixture 50 µg/ml kanamycin and 0.003% adenine hemisulfate were added. The PUUV NSs and TULV NSs mixtures were incubated at +30°C with shaking for 27 h and 20.5 h, respectively. The success of mating was verified by detecting zygote yeast cells with a microscope. Cells were collected by centrifugation at 1000×g during 10 min. Cells were resuspended in 10 ml of 0.5× YPDA/kanamycin medium (50 µg/ml).

For transformation efficiency calculations, 100 µl of 1∶10, 1∶100, 1∶1000, and 1∶10 000 dilutions were plated on SD/-Leu, SD/-Trp, and SD/-Leu/-Trp plates, and incubated at +30°C for 3–5 days. For actual screening, 200 µl of yeast cells were plated on 15-cm diameter SD/-Leu/-Trp/-His/-Ade plates, and incubated at +30°C for 9 days. Line cultures were drawn of every colony on SD/-Leu/-Trp/-His/-Ade plates and incubated at +30°C for 3–5 days.

Yeast plasmid DNA was purified by Zymoprep TM II Yeast Plasmid Miniprep (Zymo Research, Orange, CA) and used to transform *E. coli* strain DH5α competent cells. Bacterial plasmid DNA was isolated with QIAprep Spin Miniprep Kit (Qiagen, Hilden, Germany).

### Sequencing and sequencing data analysis

Plasmid DNA was sequenced using ABI PRISM^tm^ Dye Terminator sequencing kit (Perkin Elmer/ABI, Carlsbad, CA). Nucleotide and deduced protein sequences were identified using BLAST [29] and online services of Swiss-prot (http://au.expasy.org/).

### Analysis of cellular interactome

Protein interactions were searched using Cytoscape 2.8.0 program [30]. Protein interaction data for human genome were obtained from IntAct database using Pathway Commons web service [31]. In two cases data were not available for humans and *Mus musculus* was used instead (noted in Table S1). The data were further processed using the MCODE cluster finding plugin [32], the NetworkAnalyzer plugin [33] and the BiNGO gene ontology tool [34]. Gene ontology data were downloaded from the Gene Ontology project [35].

### Confocal microscopy and fluorescence resonance energy transfer (FRET) assay

Kidney cells harvested from common vole (*Microtus arvalis*), the natural host for TULV [36], were grown on coverslips and infected with TULV, strain Lodz [37], MOI 0.1 for 9 d. Cells were fixed with 4% paraformaldehyde and permeabilized with 0.1% Triton X-100 (Sigma-Aldrich, St. Louis, MO). Samples were incubated 1 h at 37°C with rabbit polyclonal antibodies against TULV NSs-GST fusion protein [38] and mouse monoclonal anti-ACBD3 antibody (Abnova, Taipei, Taiwan). Proteins were visualized using secondary antibodies tagged with Alexa Dye 546 and Alexa Dye 488 (Invitrogen, Carlsbad, CA) for NSs protein and ACBD3, respectively. Confocal microscopy and FRET assay were performed with Leica TCS SP2 microscope using 63× magnification. In FRET assay, a sample was excited with 488 nm argon laser, and emission was measured at 500–535 nm and 643–702 nm wavelengths. Then the sample was photo bleached with 561 nm DPSS laser and emission was measured again. FRET efficiency was calculated using formula: FRET_eff_ = (D_post_−D_pre_)/D_post_ for all D_post_>D_pre_, where D_post_ is intensity of donor fluorophore (green) after bleaching and D_pre_ is the intensity before bleaching.

## Results

### Controls

First, immunoblotting was performed to verify that TULV and PUUV NSs proteins were expressed from their respective plasmids. In PUUV NSs-screening the library titer was 3.4×10^7^ cells/ml, and in TULV NSs-screening 2.3×10^7^ cells/ml. In both screenings the number of clones was over one million. Both TULV NSs- and PUUV NSs-transformed yeasts expressed the NSs-fusion protein (DNA-binding domain, c-myc tag, part of MCS) properly, although PUUV NSs transformed more yeasts than TULV NSs. All samples grew well on SD/-Leu/-Trp plates as expected, so NSs- and library plasmids both expressed their genes. NSs-pACT-2 samples did not grow on SD/-Leu/-Trp/-His/-Ade plates, showing that NSs genes on their own do not activate library plasmid genes.

### Screening of mouse embryo cDNA library using the Y2H assay

In the screenings with TULV or PUUV NSs proteins as “bait”, 119/512 and 99/526 colonies, respectively, grew on line cultures. From these, nucleotide sequences encoding for 65 individual cellular proteins were identified: 21 for TULV NSs protein and 47 for PUUV NSs protein (Table S1). Three proteins were found in both groups: Acyl-CoA-binding domain containing 3 protein (ACBD3), ARP5 actin-related protein 5 homolog (ACTR5), and keratin-14 (KRT14). Three proteins from the list of potential cellular partners for TULV-NSs protein were subunits of oxidative phosphorylation complexes and were omitted from further analyses because they are known as common false positives in the Y2H screenings. One such protein appeared on the PUUV-NSs list and was omitted as well.

### Analysis of cellular interactome: proteins from the Y2H screen form an interacting network

**Figure 1:**
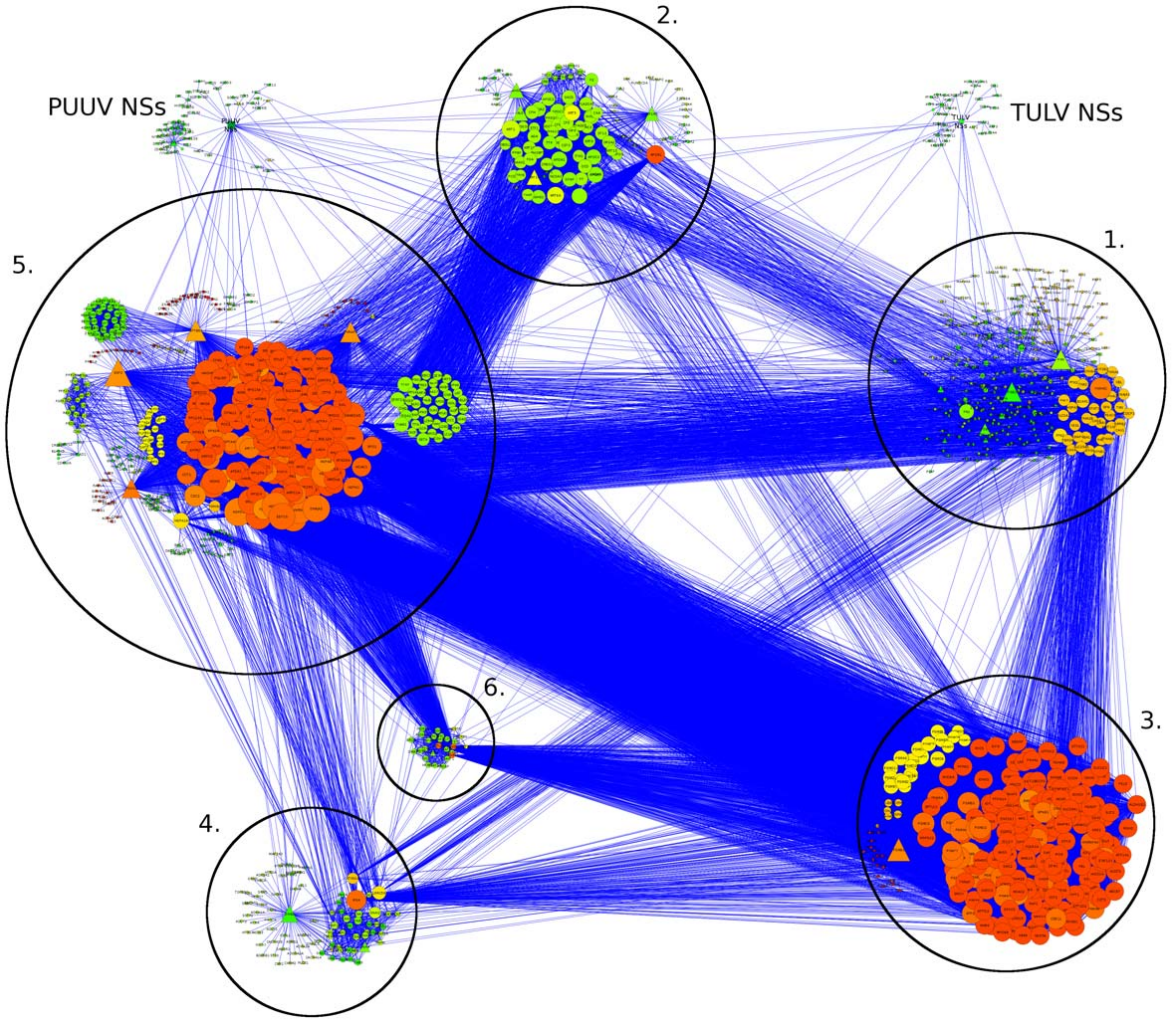
Interactome (human) of TULV and PUUV NSs proteins. Proteins of the human interactome are arranged into clusters: 1. Cellular structure, 2. Transport, 3. Proteasome, 4. Signaling, 5. Transcription & translation, 6. Histone remodeling. The size of a node is relative to the number of connections it has, the color is related to the number of connections its neighbors have (from green, few to red, many). Primary nodes are shown as triangles, secondary as circles.

The protein interaction data for the above-mentioned 65 individual cellular proteins were collected from the Pathway commons databank (see Materials and Methods). The data were available for 54 human proteins and 33 mouse *(Mus musculus)* proteins; these were visualized using the Cytoscape program yielding networks of potential protein-protein interactions. The network of human proteins consisted of 54 primary nodes connected to over 1300 secondary nodes (Figure 1). Based on the nature of shared interacting partners the majority (71%) of primary nodes were placed into one of six clusters. The clusters were named after the cellular function(s) that the majority of nodes were involved in: (1) cellular structure, (2) transport, (3) proteasome, (4) signaling, (5) transcription & translation, and (6) histone remodeling. The cores of clusters were formed by primary nodes that shared several partners (secondary nodes) with each other. Then, remaining primary nodes were assigned into a cluster if they were connected to a node already belonging to it and had function(s) similar to those of the core protein(s). From the initial clustering it was evident that, of the 55 proteins, those which could be placed into clusters shared a high level of interconnectivity, and even the nodes that could not be unequivocally placed into one of the clusters had some connections between them.

The mouse network included 33 primary and 944 secondary nodes (Figure S1), which could be in a similar fashion arranged into clusters. However, only two clusters were identified: (1) transport and (2) signaling. At this stage the deficiency of the mouse network-data became apparent. Most importantly, the interaction data for 16 out of 21 TULV-NSs- associated proteins were not available at all and the resuming five cellular proteins showed a rather low degree of connectivity. This hampered next step in the analysis (search for the intersection proteins), aimed to see what kind of relationship the primary nodes would have with each other. Therefore, the study was continued only with the human interaction data.

### Analysis of the intersection proteins

**Figure 2:**
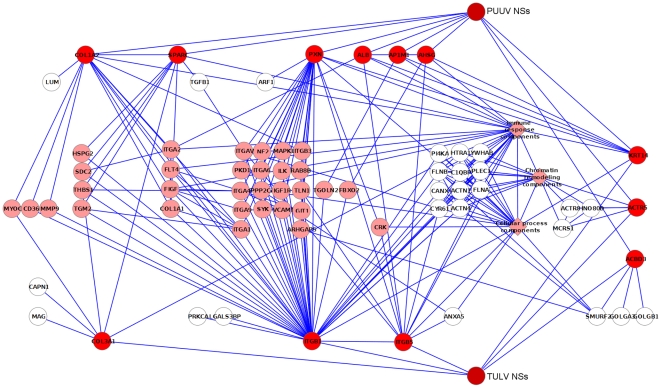
Intersection proteins. Intersection of closest shared TULV and PUUV NSs -linked nodes. Primary nodes are in red, secondary nodes in pink. Secondary nodes that do not directly connect to two different primary nodes are in white. Metanodes are represented by pink diamonds.

Since the initial grouping of primary nodes was somewhat arbitrary (several nodes could be placed into two or even more clusters), the second level of refinement was needed. It was assumed that the intersection of TULV-NSs- and PUUV-NSs-linked proteins (nodes) would be the most likely location for the cellular partner(s), because it would contain the nodes that connect directly to both bait proteins and their neighboring nodes. This intersection consisted of 12 primary nodes and 210 secondary nodes connecting them (Table S2). All 222 proteins were then further grouped based on the nodes they were connected to. On Figure 2, the primary nodes (shown as red circles) are connected directly to TULV-NSs and/or PUUV-NSs proteins while the secondary nodes are connected to the primary nodes. Note that some of the secondary nodes do not immediately connect at least two different primary nodes; these are marked white. Other secondary nodes are marked pink.

For the sake of clarity some of the nodes were condensed into three metanodes (pink diamonds) named “cellular process components”, “immune response components” and “chromatin remodeling components”. These nodes form dense, interconnected clouds that are visually distracting (metanode contents are listed under their own headings in Table S2).

Of the 12 intersecting primary nodes, only KRT14, ACTR5 and ACBD3 appeared to be directly associated with both TULV-NSs and PUUV-NSs proteins, making them the most obvious candidates. KRT14 is involved in the physical maintenance of epidermis [39]. ACTR5 is a subunit of INO80 complex that is essential for chromatin remodeling [40]. ACBD3 is involved in the maintenance of the Golgi structure and other cellular functions such as steroidogenesis [41], [42]. KRT14, together with keratin 5, are major components of the cytoskeleton of epithelial cells, maintaining cell structure and protecting it from mechanical stress [43]. Although it is known that mutations in KRT14 may increase keratinocyte susceptibility to apoptosis [44], there are no reports of its direct involvement in viral replication. Since it is an abundant protein in cell, well known to contaminate Y2H screens, KRT14 is likely to present a false-positive interaction.

Of the nine remaining proteins, one is involved in clathrin transport (AP-1 complex subunit mu-1 AP1M1), two are known to form collective tissue (collagens alpha-1 and -2 COL1A2, COL3A1), three are involved in cell adhesion (integrins beta-1 and -5 ITGB1, ITGB5, paxillin PXN) and three are secreted proteins (albumin ALB, alpha-2-HS-glycoprotein AHSG, and osteonectin SPARC). Albumin is the most common protein in blood serum and functions as a carrier for lipids and hormones. AHSG is a glycoprotein excreted from the liver and is involved in several functions, including endocytosis [45]. SPARC is found in the extracellular matrix, where it controls cell adhesion and cell cycle progression [46].

### Analysis of gene ontology (GO) data

Next, the set of 222 intersecting nodes was analyzed using BiNGO tool [34] to see which GO terms were significantly enriched. Both cellular localization and molecular function of proteins were tested, and GO data were available, respectively, for 205 and 200 proteins. For the localization study the very broad-scale GO terms (such as ‘cell surface’ or ‘cytoplasm’) were omitted, and the cut-off p-value for the GO term to be taken into consideration was set to 1E-4. Each of the remaining GO terms was a member of one of five distinct (extra)cellular locations: nucleus, cytoskeleton, vehicles, plasma membrane and extracellular space (Table 1). The proportion of GO terms that were significantly enriched varied between these locations (10–96%). Proteins located in the integrin complex were the most overexpressed (p≤1.73E-9) while the proteins in the intermediate filament of cytoskeleton were the least overexpressed (p≤1.17E-4). Interestingly, all five significantly-overexpressed nuclear proteins belonged to the histone acetyltransferase complex (i.e. to chromatin remodelling machinery).

**Table 1:** Analysis of GO data: cellular localization of intersection nodes.

GO term*	Number of nodes	Overexpression (p-value)**
**1. Cytoskeleton**	31	
Intermediate filament	11	1.17E-4
**2. Extracellular space**	24	
Plasma lipoprotein particle	5	7.08E-6
**3. Nucleus**	52	
Histone acetyltransferase complex	5	2.70E-6
**4. Plasma membrane**	64	
Basolateral plasma membrane	9	3.18E-5
Focal adhesion	7	1.11E-6
Integrin complex	9	1.73E-9
**5. Vesicle**	24	
Cytoplasmic membrane-bounded vesicle	23	6.26E-9
Pigment granule	8	6.57E-5
Secretory granule	12	6.97E-9

* GO terms follow the GO Consortium hierarchy. Higher level (generic) terms are numbered and in bold text, lower level (specific) terms are in regular text.

** The cut-off value was set to 1E-4.

The same analysis was performed with the molecular function GO data (p-value cut-off 1E-4); the results are listed in Table 2. Due to large number of rather specific GO terms some of them were grouped together under more generic GO entries. For example, the terms “cholesterol influx” and “cholesterol efflux” were listed as the “cholesterol transport”. Similarly to the first assignment, the proportion of GO terms that were significantly enriched varied between the functions (9–78%). Proteins involved in the integrin-mediated signaling were the most overexpressed (p≤2.67E-11) while the proteins involved in cell migration were the least overexpressed (p≤9.54E-4). As expected, terms related to antiviral defense response were found overexpressed in this dataset. For instance, Table 2 lists nine members of the innate immune response and six members of the complement activation process.

**Table 2:** Analysis of GO data: involvement of intersection nodes in cellular processes.

GO term*	Number of nodes	Overexpression (p-value)**
**1. Signal transduction**	71	
Integrin-mediated signaling pathway	14	2.67E-11
Transmembrane receptor protein tyrosine kinase signaling pathway	16	2.39E-7
**2. Regulation of cell growth**	18	2.00E-9
**3. Cell adhesion**	33	
Cell-matrix adhesion	11	1.06E-7
Leukocyte adhesion	5	8.36E-5
**4. Transport**	44	
Cholesterol transport	7	5.43E-7
**5. Cellular component organization and biogenesis**	54	
Chromatin modification	16	8.45E-7
Cell junction assembly	5	8.36E-5
**6. Regulation of developmental process**	32	
Regulation of apoptosis	25	1.62E-6
**7. Organ development**	40	
Ectoderm development	13	1.06E-5
**8. Response to stress**	48	
Acute inflammatory response	9	1.06E-5
Response to unfolded protein	8	2.34E-5
Blood coagulation	9	5.11E-5
Innate immune response	9	2.75E-4
**9. Regulation of signal transduction**	16	
Regulation of peptidyl-tyrosine phosphorylation	6	1.57E-4
Negative regulation of JAK-STAT cascade	3	8.21E-4
**10. Regulation of immune system process**	13	
Complement activation	6	1.78E-4
**11. Cell differentiation**	22	
Leukocyte differentiation	7	4.10E-4
**12. Cell motility**	16	
Cell migration	11	9.54E-4

* GO terms follow the GO Consortium hierarchy. Higher level (generic) terms are numbered and in bold text, lower level (specific) terms are in regular text.

** The cut-off value was set to 1E-4.

It is of interest that, with a few exceptions, proteins within a given GO term interact with each other (data not shown). For example, each of five members of histone acetyltransferase complex is known to interact with the other four proteins. Similarly, of the proteins belonging to the largest cellular process GO term group ‘regulation of apoptosis’, 16 of the 25 form an interacting network, thus suggesting that they belong to real functional units.

### Confocal microscopy and FRET assay

**Figure 3:**
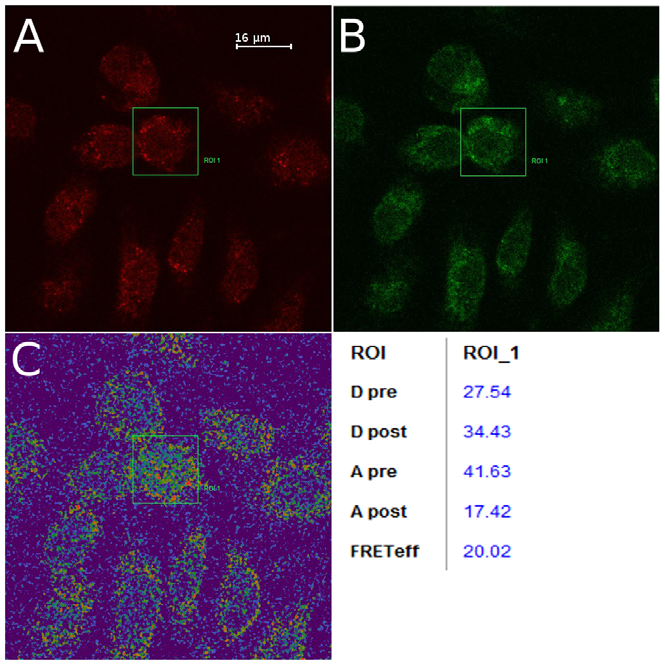
Results of FRET assay. Primary cells were infected with TULV strain Lodz for 9 days and fixed on coverslips. Cells were stained for the viral NSs protein, which was seen as bright spots around perinuclear area (A), and ACBD3 protein (B). FRET assay: D_pre_, donor intensity before bleaching, D_post_, donor intensity after bleaching, A_pre_, acceptor intensity before bleaching, A_post_, acceptor intensity after bleaching, FRET_eff_, calculated efficiency of FRET.

To prove the principle, an interaction between the viral NSs protein and the cellular ACBD3 protein was demonstrated using biochemical means. First, GST-pulldowns using GST-NSs fusion protein expressed in *E. coli* and co-immunoprecipitation with anti-NSs antibodies were attempted, but those did not yield satisfactory results. Next, confocal fluorescent microscopy and FRET assays were performed. Cells in culture were infected with TULV and proteins of interest were visualized using rabbit polyclonal anti-NSs-antibodies and mouse polyclonal anti-ACBD3-antibodies. In agreement with our earlier observations [38], the hantaviral NSs protein was seen as bright compact spots in the perinuclear area (Figure 3A), and as expected, the ACBD3 protein was located in the Golgi area (Figure 3B). Confocal microscopy revealed co-localization of the two proteins in the perinuclear area (data not shown). Interaction between NSs and ACBD3 proteins was further confirmed using FRET assay (Figure 3C). After the photo bleaching intensity of the acceptor fluorophore was significantly reduced while the intensity of donor fluorophore was slightly elevated. The efficiency of FRET assay was approximately 20%, suggesting strong interaction between the two proteins (Figure 3C).

## Discussion

In this paper, the Y2H screening for potential cellular partners of the NSs proteins of two closely related but distinct hantaviruses, TULV and PUUV, was performed and the interaction network of potential binding partners was created. The Y2H screen was performed on the mouse cDNA library and the interacting proteins were identified using genome sequence data. For the interactome analysis the human protein interaction data were used. These data are more extensive than mouse data: 65000 human interactions vs. 11800 mouse interactions listed on IntAct database, (www.pathwaycommons.org) and the difference was clearly visible in our own analysis: in the mouse database, the interaction data were available for only a small portion of TULV-NSs associated proteins (24% *vs* 76% in the human database), and the information about intracellular connections of these proteins was very limited. Even for the more numerous PUUV-NSs associated proteins the interaction data were available for a significantly smaller portion: 57% in the mouse-database *vs* 87% in the human database. Additional support for the use of human interactome data for this study comes from the fact that the mouse genome is 75% orthologous to human genome [47] and mice are widely used to model human diseases. For example, for the hantavirus infection, good correlation between pathogenicity of hantaviruses in humans and severity of a model infection in mice have been reported [48].

The proteins in the human network were shown to concentrate into six clusters that shared some interacting partners involved in different cellular functions. From this network, a subset of 12 primary nodes (proteins found in the Y2H assay) connecting 210 secondary nodes (the intersection of TULV-NSs- and PUUV-NSs-linked proteins) was selected using the Cytoscape program. These proteins are the most likely candidates for interaction(s) with the hantaviral NSs protein. Further analysis revealed statistically significant overexpression of certain GO terms, both location-wise and function-wise; allowing focusing future studies on certain cellular compartments and functional complexes. Finally, for one of the potential candidates, the ACBD3 protein, the interaction with the NSs protein was demonstrated by biochemical means, thus showing that the approach taken is useful. Biochemical analysis of interactions(s) between the hantaviral NSs protein and other potential cellular partners is on the way.

Although the Y2H assay is a well-established method for finding novel protein-protein interactions, it is not very specific and routinely produces false positives [49]. For example, cytoskeletal proteins are common contaminants and as such they are usually omitted from analysis. However, since it is known that some viruses commandeer host organelles and cellular proteins to form structures like viral factories [50], caution should be exercised when dismissing such candidates (in this case, KRT14). An additional complication is that the efficiency of the Y2H screens is not very high, and some interactions may remain undiscovered. In this study we performed two independent Y2H screenings, and observed quite large differences in the mating efficiency of yeast depending on the plasmid used. Despite this we confirmed that the transformed yeast(s) did express the NSs fusion protein properly and we were able to identify 21 (TULV) and 47 (PUUV) potential binding partners for the NSs protein. Three of these proteins were shared between both bait proteins: ACBD3, ACTR5, and KRT14. Additionally, *in silico* analysis revealed other primary nodes (proteins) located into clusters with shared secondary nodes. Whether this is due to some real, yet undiscovered, interaction or similar non-specific contaminants affecting both screenings remains to be seen, but we believe we have successfully found at least some of the relevant interaction partners for the NSs proteins.

Some of the criteria for estimating the reliability of protein-protein interactions found in the Y2H assay are reproducibility, topology and involvement in related cellular function [51]. Topology applies to the network of protein interactions, where multiple (short) paths between two interacting proteins increase the likelihood of a real *in vivo* interaction. Towards this end the interactions of hantaviral NSs and host proteins were first mapped and then rearranged into a network so that the cellular context for potential binding partners could be established. This process revealed that the majority of the cellular proteins can be grouped into clusters based on their function, and that these clusters are connected to each other (Figure 1). Some of the more interesting targets found in the Y2H screening are discussed below.

Chromatin remodeling is a necessary step for making genes accessible to transcription factors and important part of the gene expression control. INO80 chromatin remodeling complex, which in humans consists of 16 subunits, was first characterized from yeast and seems to be evolutionarily conserved [40]. It is known to be involved in transcription regulation, DNA double-stranded break repair and DNA replication. **ACTR5** is a subunit of INO80 complex that is essential for its function [52]. Interestingly, some viruses (such as RVFV), evade host innate immune response by large-scale disruption of host gene expression via chromatin remodeling [14].

Virus infection can cause morphological changes in the Golgi structure [53]. **ACBD3** (also known as Golgi complex-associated protein of 60 kDa, GCP60) is associated with giantin and golgin-160, two large structural proteins located in Golgi, and is involved in the maintenance of the Golgi structure and other cellular functions such as steroidogenesis [54] and Numb signaling [55]. In a paper by Blanc *et al.* [56] sterol metabolism was shown to be linked to the innate immune response. They found that the treatment of cells with IFN downregulates steroid biosynthesis and that has an antiviral effect. Most interestingly, recently two different groups have reported that non-structural proteins of picornaviruses interact with ACBD3 and that ACBD3 mediates the recruitment of phosphatidylinositol 4-kinase IIIβ, a host cellular factor necessary for viral RNA replication [57], [58]. This suggests that the ACBD3 can play a previously unknown role in the host immune response. In our experiments, ACBD3 was found in association with the hantaviral NSs protein (Figure 3); this interaction is the subject of an ongoing investigation.

Several viruses localize to the Golgi apparatus during their replication cycle, typically for assembly of viral particles and budding to cell surface. AP-1 is an adaptor protein complex that regulates sorting of vesicles between endosomes and trans-Golgi structure by linking clathrin to coated vesicles. **AP1M1** is a subunit of this complex that regulates its cycling between membrane and cytoplasm [59]. Endocytosis is a major way of entering into host cells for viruses, and although NSs protein is expressed only after viral entry the AP1M1 protein is nevertheless an interesting target because of its role in protein transport both in and out of cells.

The trans-Golgi network protein 2 (**TGOLN2**) is a membrane protein that cycles between trans-Golgi structure and cell surface and associates with clathrin adaptor AP2 [60] and integrin beta 1 [61]. It might be involved in transport of coagulation factor XIII-A in macrophages [62], but otherwise its function(s) are still largely unknown.

Fetuin-A (**AHSG**), as previously mentioned, is a serum glycoprotein with several functions, including induction of low-level inflammation [63] and antagonism of transforming growth factor beta (TGF-β), a widely produced cytokine [64]. Although AHSG is involved in the inflammation mechanism, there have not been reports on its direct involvement in virus infection. Additionally, it has been reported that in patients with viral hepatitis there was no significant decrease of serum AHSG levels [65].

SMAD-specific E3 ubiquitin protein ligase 2 (**SMURF2**) is involved in the regulation of TGF-β by marking Smad signal transducer proteins for proteasomal degradation by ubiquitination [66]. Although Smads are best known to regulate TGF-β, they have other functions, such as Smad3 which binds to RNA splicing complex [67].

**CRK adaptor proteins** are involved in several signaling cascades in the cell. They contain several SH2 and SH3 (src-homology) domains that bind tyrosine-phosphorylated cytosolic proteins [68]. They are known to bind to NS1 protein of the influenza A virus, both the 1918 pandemic strain [69] and the more recent avian influenza [70].

To conclude, this study revealed good overlapping of the two sets of primary and especially secondary targets (cellular partners) for the hantaviral NSs protein. These potential cellular counterparts are involved, for example, in chromatin remodeling, signaling and also in Golgi structure and functioning. In our *in vitro* experiments ACBD3 was found in association with hantaviral NSs protein; this and other potential interactions are the subject of ongoing investigation. So far, no evidence for the interaction of hantaviral NSs protein with known components of IFN induction and signaling cascades (such as RIG-I or MDA5) was found suggesting that these interactions are, perhaps, mediated by other proteins.

As stated in the introduction, different bunyaviruses use different strategies to deal with the host cell defence such as an innate immunity response. The list that includes 12 primary and 210 secondary nodes presented in this paper does not contain any of the known cellular partners for bunyaviral NSs proteins thus confirming this point and urging to look for novel mechanisms of counteraction. It should not be forgotten than the NSs proteins might well be multifunctional and perform other duties in a virus replication cycle.

## Supporting Information

Figure S1 **Interactome (mouse) of TULV and PUUV NSs proteins.** Each protein of the mouse interactome is represented by a node. The size of a node is relative to the number of connections it has, and its color is related to the number of connections its neighbors have (from green, few to red, many).
(TIF)

Table S1 **Proteins found in the Y2H screening.**
(DOC)

Table S2 **Proteins found in the intersection of TULV-NSs- and PUUV-NSs-linked nodes.**
(DOC)
