# Supplementary figures and images for: Searching for Cellular Partners of Hantaviral Nonstructural Protein NSs: Y2H Screening of Mouse cDNA Library and Analysis of Cellular Interactome
Source: PLoS One. 2012 Apr 10;7(4):e34307. doi: 10.1371/journal.pone.0034307 (PMC3323627; doi:10.1371/journal.pone.0034307)

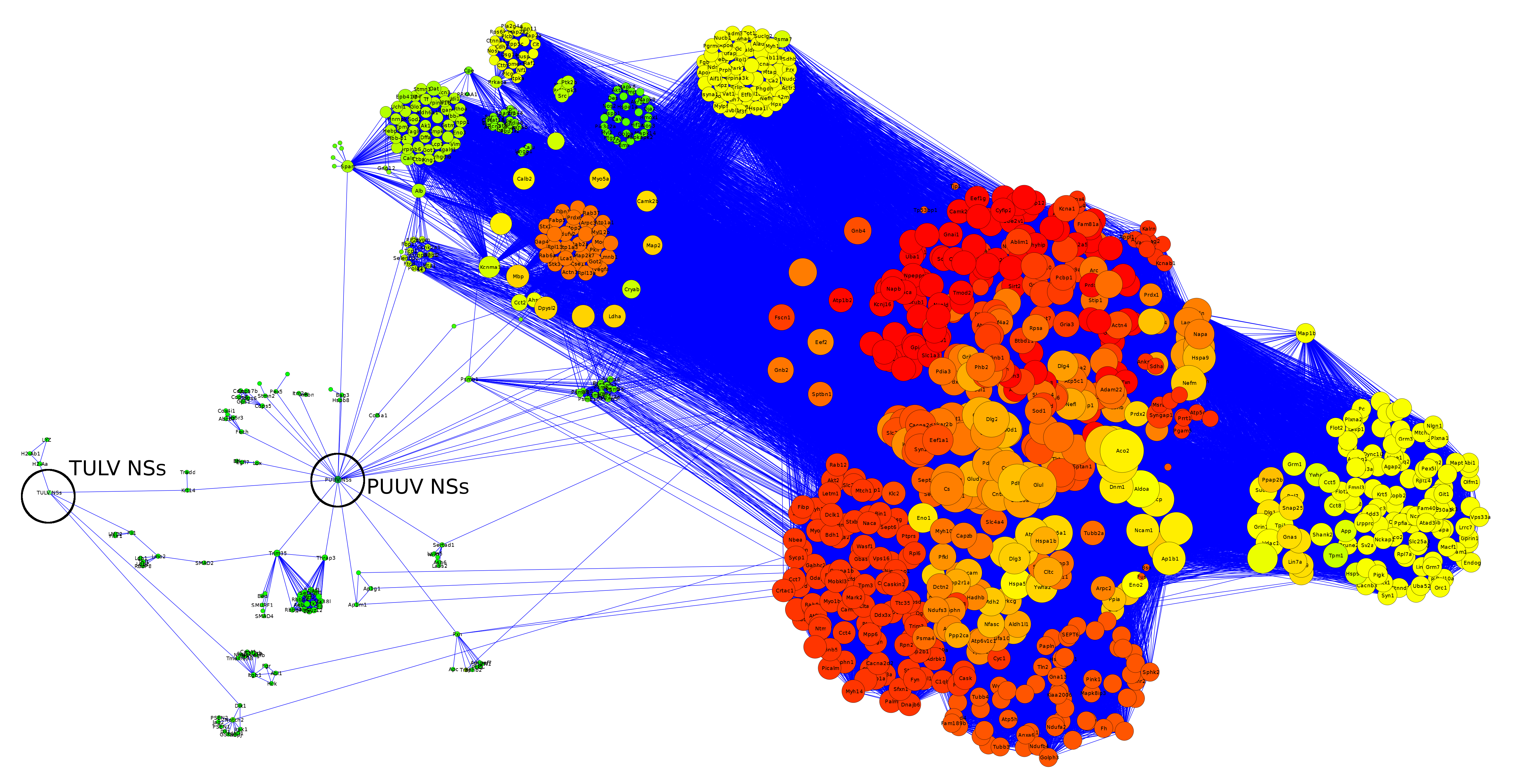

Supplement: Figure S1 — Interactome (mouse) of TULV and PUUV NSs proteins. Each protein of the mouse interactome is represented by a node. The size of a node is relative to the number of connections it has, and its color is related to the number of connections its neighbors have (from green, few to red, many). (TIF) [file pone.0034307.s001.tif]
