# Supplementary material for: Searching for Cellular Partners of Hantaviral Nonstructural Protein NSs: Y2H Screening of Mouse cDNA Library and Analysis of Cellular Interactome
Source: PLoS One. 2012 Apr 10;7(4):e34307. doi: 10.1371/journal.pone.0034307 (PMC3323627; doi:10.1371/journal.pone.0034307)
Supplement: Table S1 — Proteins found in the Y2H screening. (DOC) [file pone.0034307.s002.doc]

**Supplementary Table 1. Proteins found in the Y2H screening**

| **1. Nodes shared by both bait proteins** | **Gene name** | **Copy number*** | **RefSeq accession number** | **Cluster**** |
| --- | --- | --- | --- | --- |
| Acyl-CoA-binding domain-containing protein 3 | ACBD3 | 10 | NP_073572.2 | 0*** |
| Actin-related protein 5 | ACTR5 | 2 | NP_079131.3 | 6 |
| Keratin 14 | KRT14 | 2 | NP_000517.2 | 2 |
|  |  |  |  |  |
| **2. Nodes related to Tula NSs protein** | **Gene name** | **Copy number** | **RefSeq accession number** | **Cluster** |
| ADP-ribosylation factor interacting protein 1 | ARFIP1 | 1 | NP_001020766.1 | 0 |
| CAMP responsive element binding protein 3-like 2 | CREB3L2 | 1 | NP_919047.2 | 0 |
| Collagen, type III, alpha 1 | COL3A1 | 1 | NP_000081.1 | 1 |
| Delta-like 1 homolog | DLK1 | 2 | NP_003827.3 | 0 |
| Deoxyhypusine hydroxylase / monooxygenase | DOHH | 1 | NP_001138637.1 | 0 |
| Desmocollin-2 | DSC2 | 2 | NP_004940.1 | 1 |
| Differentially expressed in FDCP 8 | DEF8 | 1 | NP_473387.1 | n.a.# |
| DnaJ homolog subfamily C member 21 | DNAJC21 | 1 | NP_919259.3 | n.a. |
| Dystrophia myotonica-containing WD repeat motif | DMWD | 1 | NP_004934.1 | 2 |
| Friend leukemia virus integration 1 | FLI1 | 1 | NP_002008.2 | 5 |
| H2-Aa histocompatibility 2 class II antigen A alpha | H2-Aa | 1 | NP_034508.2 | 0 |
| High-mobility group nucleosomal binding domain 2 | HMGN2 | 2 | NP_005508.2 | 5 |
| Integrin beta-1 precursor | ITGB1 | 34 | NP_002202.2 | 1 |
| Integrin beta-5 precursor | ITGB5 | 1 | NP_002204.2 | 1 |
| Matrix metalloproteinase-14 | MMP-14 | 5 | NP_004986.1 | 1 |
| RING finger protein 182 | RNF182 | 6 | NP_001158504.1 | n.a. |
| Solute carrier family 30 (zinc transporter), member 1 | SLC30A1 | 2 | NP_067017.2 | n.a. |
| Stearoyl-Coenzyme A desaturase 2 | SCD2 | 1 | NP_033154.2 | n.a. |
|  |  |  |  |  |
| **3. Nodes related to Puumala NSs protein** | **Gene name** | **Copy number** | **RefSeq accession number** | **Cluster** |
| Adaptor-related protein complex 1, mu 1 subunit | AP1M1 | 1 | NP_001123996.1 | 2 |
| Alpha-2-HS-glycoprotein precursor | AHSG | 2 | NP_001613.2 | 2 |
| Alpha-fetoprotein | AFP | 3 | NP_001125.1 | 0 |
| Amine oxidase, copper containing 2 (retina-specific) | AOC2 | 1 | NP_033720.2 | 0 |
| BCL-2-associated athanogene 3 | BAG3 | 1 | NP_004272.2 | 5 |
| Carboxypeptidase E precursor | CPE | 1 | NP_001864.1 | 2 |
| Cathepsin E precursor | CTSE | 1 | NP_001901.1 | 0 |
| Clathrin heavy chain 1 | CLTC | 1 | NP_004850.1 | 6 |
| Collagen alpha-1 type V precursor | COL5A1 | 1 | NP_000084.3 | 1 |
| Collagen alpha-2 type I precursor | COL1A2 | 13 | NP_000080.2 | 1 |
| Collectin sub-family member 12 | COLEC12 | 1 | NP_569057.1 | n.a. |
| COP9 signalosome complex subunit 5 | COPS5 | 1 | NP_006828.2 | 5 |
| DNAJ homolog subfamily B member 4 | DNAJB4 | 7 | NP_008965.2 | 4 |
| Elongation factor 2 | EEF2 | 1 | NP_001952.1 | 5 |
| Family with sequence similarity 96 member B | FAM96B | 1 | NP_057146.1 | 0 |
| Fascin homolog 1 | FSCN1 | 1 | NP_003079.1 | 5 |
| Ferrochelatase, mitochondrial precursor | FECH | 1 | NP_000131.2 | 0 |
| G protein subunit beta-2 | GNB2 | 1 | NP_005264.2 | 4 |
| G protein subunit beta-4 | GNB4 | 1 | NP_067642.1 | 4 |
| Integral membrane protein 2A (*Mus musculus*) | Itm2A | 1 | NP_032435.2 | 0 |
| Lysyl oxidase | LOX | 1 | NP_002308.2 | 0 |
| MAM domain-containing protein 4 | MAMDC4 | 1 | NP_996803.2 | n.a. |
| Mesoderm-specific transcript homolog | MEST | 1 | NP_002393.2 | 0 |
| Microtubule-associated protein 1B | MAP1B | 2 | NP_005900.2 | 1 |
| Myosin regulatory light chain 2, ventricular/cardiac muscle isoform | MYL2 | 1 | NP_034991.3 | 1 |
| Na+/K+ -ATPase beta 2 subunit | ATP1B2 | 1 | NP_001669.3 | 0 |
| Nuclear receptor-binding protein 1 | NRBP1 | 1 | NP_037524.1 | 2 |
| Paxillin | PXN | 1 | NP_001074324.1 | 1 |
| Peroxisomal biogenesis factor 5 | PEX5 | 1 | NP_001124495.1 | 5 |
| Phenazine biosynthesis-like protein domain containing | PBLD | 1 | NP_071412.2 | 0 |
| Proteasome subunit beta type-1 | PSMB1 | 1 | NP_002784.1 | 3 |
| Protocadherin gamma subfamily A, 4 | PCDHGA4 | 1 | NP_061740.1 | n.a. |
| Secreted protein acidic and rich in cysteine (Osteonectin) | SPARC | 1 | NP_003109.1 | 1 |
| SERTA domain-containing protein 1 | SERTAD1 | 1 | NP_037508.2 | 5 |
| Serum albumin precursor | ALB | 2 | NP_000468.1 | 2 |
| Spectrin beta chain, non-erythroid 1 | SPTBN1 | 2 | NP_003119.2 | 5 |
| Stathmin-like 2 | STMN2 | 1 | NP_008960.2 | 5 |
| Synaptophysin-like 1 | SYPL1 | 3 | NP_006745.1 | 0 |
| Tetraspanin 18 | TSPAN18 | 1 | NP_570139.3 | n.a. |
| Thyroid hormone receptor associated protein 3 | THRAP | 1 | NP_005110.2 | 5 |
| Transient receptor potential cation channel subfamily M member 5 | TRPM5 | 1 | NP_055370.1 | n.a. |
| Tripartite motif-containing protein 35 (*Mus musculus*) | Trim35 | 1 | NP_084255.2 | 5 |
| Troponin T type 2 (cardiac) | TNNT2 | 1 | NP_000355.2 | 5 |
| Wilms tumor 1 interacting protein | WTIP | 1 | NP_001073905.1 | n.a. |

* Copy number, the number of clones found in screenings.

** Cluster refers to protein location in Figure 1

*** Not located in any cluster

# Interaction data are not available.
