# Supplementary material for: Searching for Cellular Partners of Hantaviral Nonstructural Protein NSs: Y2H Screening of Mouse cDNA Library and Analysis of Cellular Interactome
Source: PLoS One. 2012 Apr 10;7(4):e34307. doi: 10.1371/journal.pone.0034307 (PMC3323627; doi:10.1371/journal.pone.0034307)
Supplement: Table S2 — Proteins found in the intersection of TULV-NSs- and PUUV-NSs-linked nodes. (DOC) [file pone.0034307.s003.doc]

**Supplementary Table 2. Proteins found in the intersection of TULV-NSs- and PUUV-NSs-linked nodes**

| **Primary nodes** |  |
| --- | --- |
| ACBD3 | Golgi resident protein GCP60 |
| ACTR5 | Actin-related protein 5 |
| AHSG | Alpha-2-HS-glycoprotein |
| ALB | Serum albumin |
| AP1M1 | AP-1 complex subunit mu-1 |
| COL1A2 | Collagen alpha-2(I) chain |
| COL3A1 | Collagen alpha-1(III) chain |
| ITGB1 | Integrin beta-1 |
| ITGB5 | Integrin beta-5 |
| KRT14 | Keratin, type I cytoskeletal 14 |
| PXN | Paxillin |
| SPARC | SPARC |
| **Secondary nodes** |  |
| ACTN1 | Alpha-actinin-1 |
| ACTN4 | Alpha-actinin-4 |
| ACTR8 | Actin-related protein 8 |
| ANXA5 | Annexin A5 |
| ARF1 | ADP-ribosylation factor 1 |
| ARHGAP5 | Rho GTPase-activating protein 5 |
| C1QBP | Complement component 1 Q subcomponent-binding protein, mitochondrial |
| CANX | Calnexin |
| CAPN1 | Calpain-1 catalytic subunit |
| CD36 | Platelet glycoprotein 4 |
| COL1A1 | Collagen alpha-1(I) chain |
| CRK | Proto-oncogene C-crk |
| CYR61 | Protein CYR61 |
| FBXO2 | F-box only protein 2 |
| FIGF | Vascular endothelial growth factor D |
| FLNA | Filamin-A |
| FLNB | Filamin-B |
| FLT4 | Vascular endothelial growth factor receptor 3 |
| GIT1 | ARF GTPase-activating protein GIT1 |
| GOLGA3 | Golgin subfamily A member 3 |
| GOLGB1 | Golgin subfamily B member 1 |
| HSPG2 | Basement membrane-specific heparan sulfate proteoglycan core protein |
| HTRA1 | Serine protease HTRA1 |
| IGF1R | Insulin-like growth factor 1 receptor |
| ILK | Integrin-linked protein kinase |
| INO80D | INO80 complex subunit D |
| ITGA1 | Integrin alpha-1 |
| ITGA2 | Integrin alpha-2 |
| ITGA4 | Integrin alpha-4 |
| ITGA6 | Integrin alpha-6 |
| ITGA9 | Integrin alpha-9 |
| ITGAV | Integrin alpha-V |
| ITGB3 | Integrin beta-3 |
| LGALS3BP | Galectin-3-binding protein |
| LUM | Lumican |
| MAG | Myelin-associated glycoprotein |
| MAPK1 | Mitogen-activated protein kinase 1 |
| MCRS1 | Microspherule protein 1 |
| MMP9 | Matrix metalloproteinase-9 |
| MYOC | Myocilin |
| NF2 | Merlin |
| PI4KA | Phosphatidylinositol 4-kinase alpha |
| PKD1 | Polycystin-1 |
| PLEC1 | Plectin-1 |
| PPP2CA | Serine/threonine-protein phosphatase 2A catalytic subunit alpha isoform |
| PRKCA | Protein kinase C alpha type |
| RAB8B | Ras-related protein Rab-8B |
| SDC2 | Syndecan-2 |
| SMURF2 | E3 ubiquitin-protein ligase SMURF2 |
| SYK | Tyrosine-protein kinase SYK |
| TGFB1 | Transforming growth factor beta-1 |
| TGM2 | Protein-glutamine gamma-glutamyltransferase 2 |
| TGOLN2 | Trans-Golgi network integral membrane protein 2 |
| THBS1 | Thrombospondin-1 |
| TLN1 | Talin-1 |
| VCAM1 | Vascular cell adhesion protein 1 |
| YWHAB | 14-3-3 protein beta/alpha |
| **Metanode: Chromatin remodeling components** |  |
| ACTL6A | Actin-like protein 6A |
| ACTR6 | Actin-related protein 6 |
| BRD8 | Bromodomain-containing protein 8 |
| DMAP1 | DNA methyltransferase 1-associated protein 1 |
| EAF1 | ELL-associated factor 1 |
| EPC1 | Enhancer of polycomb homolog 1 |
| EPC2 | Enhancer of polycomb homolog 2 |
| ERVK6 | HERV-K_19q12 provirus ancestral Pol protein |
| GATS | Putative protein GATS |
| HTATIP | Histone acetyltransferase HTATIP |
| ING3 | Inhibitor of growth protein 3 |
| INO80 | Putative DNA helicase INO80 complex homolog 1 |
| INO80B | INO80 complex subunit B |
| INO80E | INO80 complex subunit E |
| MORF4L1 | Mortality factor 4-like protein 1 |
| MRGBP | MRG-binding protein |
| RUVBL1 | RuvB-like 1 |
| RUVBL2 | RuvB-like 2 |
| SRCAP | Helicase SRCAP |
| TCF3 | Transcription factor E2-alpha |
| VPS72 | Vacuolar protein sorting-associated protein 72 homolog |
| YEATS4 | YEATS domain-containing protein 4 |
| ZNHIT1 | Zinc finger HIT domain-containing protein 1 |
| **Metanode:**  **Cellular process components** |  |
| ATP2A2 | Sarcoplasmic/endoplasmic reticulum calcium ATPase 2 |
| ATXN10 | Ataxin-10 |
| BCAR1 | Breast cancer anti-estrogen resistance protein 1 |
| BCAR3 | Breast cancer anti-estrogen resistance protein 3 |
| CAV1 | Caveolin-1 |
| CDC2 | Cell division control protein 2 homolog |
| CDCP1 | CUB domain-containing protein 1 |
| CKAP4 | Cytoskeleton-associated protein 4 |
| CSE1L | Exportin-2 |
| CTNNA1 | Catenin alpha-1 |
| CTNND1 | Catenin delta-1 |
| DNAJA1 | DnaJ homolog subfamily A member 1 |
| DNAJA2 | DnaJ homolog subfamily A member 2 |
| DNAJA3 | DnaJ homolog subfamily A member 3, mitochondrial |
| EEF1A1 | Elongation factor 1-alpha 1 |
| EFNB1 | Ephrin-B1 |
| EGFR | Epidermal growth factor receptor |
| EIF2B3 | Translation initiation factor eIF-2B subunit gamma |
| EPHA2 | Ephrin type-A receptor 2 |
| EPHB4 | Ephrin type-B receptor 4 |
| ERBB2 | Receptor tyrosine-protein kinase erbB-2 |
| FHL2 | Four and a half LIM domains protein 2 |
| GRB2 | Growth factor receptor-bound protein 2 |
| HIST1H1E | Histone H1.4 |
| HIST1H2BC | Histone H2B type 1-C/E/F/G/I |
| HNRNPF | Heterogeneous nuclear ribonucleoprotein F |
| HNRNPH1 | Heterogeneous nuclear ribonucleoprotein H |
| HNRNPK | Heterogeneous nuclear ribonucleoprotein K |
| HSP90AB1 | Heat shock protein HSP 90-beta |
| HSPA1A | Heat shock 70 kDa protein 1 |
| HSPA5 | 78 kDa glucose-regulated protein |
| HSPA8 | Heat shock cognate 71 kDa protein |
| HSPA9 | Stress-70 protein, mitochondrial |
| HSPB1 | Heat shock protein beta-1 |
| HSPD1 | 60 kDa heat shock protein, mitochondrial |
| KPNB1 | Importin subunit beta-1 |
| KRT17 | Keratin, type I cytoskeletal 17 |
| LRRC59 | Leucine-rich repeat-containing protein 59 |
| PEX19 | Peroxisomal biogenesis factor 19 |
| PKP2 | Plakophilin-2 |
| PTK2 | Focal adhesion kinase 1 |
| PTK2B | Protein tyrosine kinase 2 beta |
| S100A11 | Protein S100-A11 |
| SFN | 14-3-3 protein sigma |
| SHC1 | SHC-transforming protein 1 |
| SLC25A3 | Phosphate carrier protein, mitochondrial |
| SLC25A5 | ADP/ATP translocase 2 |
| SLC3A2 | 4F2 cell-surface antigen heavy chain |
| STAT3 | Signal transducer and activator of transcription 3 |
| TBRG4 | Protein TBRG4 |
| TNK2 | Activated CDC42 kinase 1 |
| TRIM29 | Tripartite motif-containing protein 29 |
| TUBA1A | Tubulin alpha-1A chain |
| TUBB | Tubulin beta chain |
| TXN | Thioredoxin |
| UBB | Ubiquitin |
| XPOT | Exportin-T |
| YES1 | Proto-oncogene tyrosine-protein kinase Yes |
| YWHAE | 14-3-3 protein epsilon |
| YWHAQ | 14-3-3 protein theta |
| YWHAZ | 14-3-3 protein zeta/delta |
| **Metanode:**  **Immune response components** |  |
| AGA | N(4)-(beta-N-acetylglucosaminyl)-L-asparaginase |
| AMPD3 | AMP deaminase 3 |
| APOA1 | Apolipoprotein A-I |
| APOA2 | Apolipoprotein A-II |
| APOA4 | Apolipoprotein A-IV |
| APOC1 | Apolipoprotein C-I |
| APOC2 | Apolipoprotein C-II |
| APOC3 | Apolipoprotein C-III |
| C4A | Complement C4-A |
| CFB | Complement factor B |
| CFD | Complement factor D |
| CFH | Complement factor H |
| CHKB | Choline/ethanolamine kinase |
| CRB1 | Crumbs homolog 1 |
| CST3 | Cystatin-C |
| CTAGE5 | Cutaneous T-cell lymphoma-associated antigen 5 |
| DCD | Dermcidin |
| DERL1 | Derlin-1 |
| ETF1 | Eukaryotic peptide chain release factor subunit 1 |
| EXOC6 | Exocyst complex component 6 |
| F2 | Prothrombin |
| F7 | Coagulation factor VII |
| FAM71E2 | Uncharacterized protein DKFZp434G1729 |
| FGA | Fibrinogen alpha chain |
| FN1 | Fibronectin |
| GFAP | Glial fibrillary acidic protein |
| GSN | Gelsolin |
| HPX | Hemopexin |
| IGDCC4 | Immunoglobulin superfamily DCC subclass member 4 |
| IGHG1 | Ig gamma-1 chain C region |
| IGKC | Ig kappa chain C region |
| IGKV2-40 | Ig kappa chain V-II region Cum |
| IGL@ | IGL@ protein |
| ITIH1 | Inter-alpha-trypsin inhibitor heavy chain H1 |
| KIAA0232 | Uncharacterized protein KIAA0232 |
| KRT1 | Keratin, type II cytoskeletal 1 |
| KRT10 | Keratin, type I cytoskeletal 10 |
| KRT13 | Keratin, type I cytoskeletal 13 |
| KRT16 | Keratin, type I cytoskeletal 16 |
| KRT5 | Keratin, type II cytoskeletal 5 |
| KRT6A | Keratin, type II cytoskeletal 6A |
| KRT6B | Keratin, type II cytoskeletal 6B |
| KRT9 | Keratin, type I cytoskeletal 9 |
| NCOA3 | Nuclear receptor coactivator 3 |
| OBSL1 | Obscurin-like protein 1 |
| OR3A2 | Olfactory receptor 3A2 |
| OR8D2 | Olfactory receptor 8D2 |
| PCDH1 | Protocadherin-1 |
| PF4V1 | Platelet factor 4 variant |
| PHC3 | Polyhomeotic-like protein 3 |
| PLA2G4F | PLA2G4F protein |
| PPBP | Platelet basic protein |
| PRSS3 | Trypsin-3 |
| QTRTD1 | Queuine tRNA-ribosyltransferase domain-containing protein 1 |
| RANBP2 | E3 SUMO-protein ligase RanBP2 |
| RYR2 | Ryanodine receptor 2 |
| SACS | Sacsin |
| SCAF1 | Splicing factor, arginine/serine-rich 19 |
| SERPING1 | Plasma protease C1 inhibitor |
| SETX | Probable helicase senataxin |
| SH3BP5 | SH3 domain-binding protein 5 |
| ST13 | Hsc70-interacting protein |
| TIAM1 | T-lymphoma invasion and metastasis-inducing protein 1 |
| TLN2 | Talin-2 |
| TRY6 | Putative trypsin-6 |
| TSC22D1 | TSC22 domain family protein 1 |
| TTPAL | Alpha-tocopherol transfer protein-like |
| TTR | Transthyretin |
| ZNF292 | Zinc finger protein 292 |
